# Histone Modulation Blocks Treg-Induced Foxp3 Binding to the IL-2 Promoter of Virus-Specific CD8^+^ T Cells from Feline Immunodeficiency Virus-Infected Cats

**DOI:** 10.3390/v10060287

**Published:** 2018-05-27

**Authors:** Mukta Nag, Yan Wang, Kristina De Paris, Jonathan E. Fogle

**Affiliations:** 1 Department of Population Health and Pathobiology, College of Veterinary Medicine, North Carolina State University, 1060 William Moore Drive, Raleigh, NC 27607, USA; mnag@ncsu.edu; 2 Department of Microbiology and Immunology, University of North Carolina at Chapel Hill, Chapel Hill, NC 27599, USA; ywang102@email.unc.edu (Y.W.); kristina_abel@med.unc.edu (K.D.P.)

**Keywords:** Feline Immunodeficiency Virus, CD8^+^ T cells, CD8^+^ T cell dysfunction, T regulatory cells, Treg suppression, Foxp3, IL-2 suppression, epigenetics, histone acetylation

## Abstract

CD8^+^ T cells are critical for controlling HIV infection. During the chronic phase of lentiviral infection, CD8^+^ T cells lose their proliferative capacity and exhibit impaired antiviral function. This loss of CD8^+^ T cell function is due, in part, to CD4^+^CD25^+^ T regulatory (Treg) cell-mediated suppression. Our research group has demonstrated that lentivirus-activated CD4^+^CD25^+^ Treg cells induce the repressive transcription factor forkhead box P3 (Foxp3) in autologous CD8^+^ T cells following co-culture. We have recently reported that Treg-induced Foxp3 binds the interleukin-2 (IL-2), interferon-γ (IFN- γ), and tumor necrosis factor-α (TNF-α) promoters in virus-specific CD8^+^ T cells. These data suggest an important role of Foxp3-mediated CD8^+^ T cell dysfunction in lentiviral infection. To elucidate the mechanism of this suppression, we previously reported that decreased methylation facilitates Foxp3 binding in mitogen-activated CD8^+^ T cells from feline immunodeficiency virus (FIV)-infected cats. We demonstrated the reduced binding of Foxp3 to the IL-2 promoter by increasing methylation of CD8^+^ T cells. In the studies presented here, we ask if another form of epigenetic modulation might alleviate Foxp3-mediated suppression in CD8^+^ T cells. We hypothesized that decreasing histone acetylation in virus-specific CD8^+^ T cells would decrease Treg-induced Foxp3 binding to the IL-2 promoter. Indeed, using anacardic acid (AA), a known histone acetyl transferase (HAT) inhibitor, we demonstrate a reduction in Foxp3 binding to the IL-2 promoter in virus-specific CD8^+^ T cells co-cultured with autologous Treg cells. These data identify a novel mechanism of Foxp3-mediated CD8^+^ T cell dysfunction during lentiviral infection.

## 1. Introduction

Acquired Immunodeficiency Syndrome (AIDS) lentiviral infections such as Human Immunodeficiency Virus (HIV) and Feline Immunodeficiency Virus (FIV) infection are marked by robust expansion of CD8^+^ T cells during the acute phase which is followed by a steady state lower level of virus-specific CD8^+^ T cells during the chronic phase of infection. CD8^+^ T cells in the chronic phase of infection are characterized by impaired proliferative capacity, reduced cytolytic function and dysfunctional effector function [1,2,3]. Interleukin-2 (IL-2) signals are important for the differentiation and homeostasis of various lymphocyte subsets including CD8^+^ T cells. Naive CD8^+^ T cells undergo rapid expansion and differentiation based on the strength and duration of IL-2 signals into either short-lived effector T cells or long lived memory T cells [4,5,6]. Antigen-specific CD8^+^ T cells respond to autocrine and paracrine IL-2 signals via the high affinity trimer IL-2-R to expand and differentiate to mount a potent immune response [7]. During chronic infection, virus-specific CD8^+^ T cells show a decrease and then loss of IL-2 expression along with progressive loss of antiviral cytokines such as interferon-γ (IFN-γ) and tumor necrosis factor-α (TNF-α) [8,9]. The accumulation of dysfunctional CD8^+^ T cells contributes to viral persistence and is a major obstacle to HIV cure strategies. However, recent studies suggest these dysfunctional CD8^+^ T cells may be rescued and contribute to the elimination of viral reservoirs. For example, it was reported that latently-infected CD4^+^ T cells could not be cleared by CD8^+^ T cells after virus reactivation with latency reversal drugs unless stimulated with gag-1 peptide [10]. Similarly, another study reported that priming dysfunctional CD8^+^ T cells restored CD8^+^ T cell function, leading to elimination of HIV reservoirs [11]. 

CD4^+^ CD25^+^ T regulatory (Treg) cells are progressively activated during FIV and HIV infection and suppress the proliferation and effector functions of both CD4^+^ and CD8^+^ T cells [12,13,14,15]. CD4^+^ CD25^+^ Treg cells stably express high levels of the transcription factor Forkhead Box P3 (Foxp3) that is critical and sufficient for the development and suppressive function of Treg cells [16,17,18,19]. Using the FIV model for AIDS lentiviral persistence, our investigations focus upon the interaction between lentivirus-activated CD4^+^CD25^+^ Treg cells and CD4^+^ and CD8^+^ target cells. Specifically, our aim is to define the molecular events that contribute to dysfunction in virus specific T cells, following their interaction with autologous Treg cells [20,21,22]. Treg cells from FIV+ cats exhibit heightened suppressor function when compared to Treg cells from uninfected control cats [23,24]. This Treg-mediated suppression of CD4^+^ and CD8^+^ T cells is tumor growth factor-β (TGF-β) dependent. Membrane-bound TGF-β expressed on FIV-activated Treg cells ligates tumor growth factor-β receptor-II (TGF-βRII) displayed on the surface of target effector cells [20,25]. This ligation leads to Smad phosphorylation, contributing to Foxp3 induction in effector cells [20,25]. Previously, we have demonstrated that the induction of Foxp3 in CD4^+^ T helper cells leads to the conversion of these cells into “induced” Treg cells that exhibit Treg cell phenotype and function [26]. 

Foxp3 is known to bind to the IL-2 and IFN-γ promoters and suppress transcription of these important cytokines [17,27]. Transient expression of Foxp3 in activated CD8^+^ T cells is most likely a mechanism to control the CD8^+^ T cell response during FIV/ HIV infection [23,28]. However, during FIV infection, TGF-β—TGF-βRII binding in activated CD8^+^ T cells can induce stable Foxp3 expression [20,25]. We have demonstrated that Foxp3 binds the IL-2 promoter in virus-nonspecific CD8^+^ T cells following Treg cell/CD8^+^ T cell co-culture [21]. More recently, we have demonstrated Foxp3 binding to the IL-2, IFN-γ and TNF-α promoter regions in virus-specific CD8^+^ T cells from FIV+ cats (and not FIV-control cats) following Treg cell co-culture [22]. Although our focus is on the repressor function of Foxp3 in this manuscript, it is important to note that Foxp3 can act as an activator as well in a context-dependent manner [29,30]. 

Epigenetic modifications are heritable alterations in the genome that are not due to changes in the DNA sequence. These molecular changes, e.g., DNA methylation, post-translational histone methylation and histone acetylation, along with chromatin remodeling, alter DNA accessibility and chromatin structure, thereby changing gene expression [31,32,33,34]. Genes poised for active transcription have in general, hypo-methylated DNA and possess hyper-acetylated histones in their promoters. Virus-specific CD8^+^ T cells actively transcribe genes essential to differentiation and antiviral function and thus exhibit an “open” chromatin conformation at essential promoter regions. At the same time, this “open” chromatin formation allows repressive transcription factors such as Foxp3 to bind to IL-2, TNF-α, and IFN-γ promoter regions and cause suppression of these genes [21,22]. Epigenetic modifications including de-methylated DNA and acetylated histones H3 and H4 have been reported for several chronic viral infections, such as lymphocytic choriomeningitis virus (LCMV), FIV, and HIV [21,35,36,37,38]. These epigenetic modifications are, however, reversible. We demonstrated that hyper-methylating mitogen-activated CD8^+^ T cells prior to autologous Treg co-culture blocked Foxp3 binding to the IL-2 promoter in CD8^+^ T cells [21]. Based upon these findings, we asked if promoting histone de-acetylation, could be utilized as another means to prevent Treg cell-induced Foxp3 binding to the IL-2 promoter in virus-specific CD8^+^ T cells.

Anacardic acid is a plant based bioactive compound isolated from the *Anacardium occidentale* (cashew nut) shell which is structurally similar to salicylic acid [39,40]. Anacardic acid inhibits p300 histone acetyltransferase (HAT) and the p300/cyclic adenosine monophosphate (AMP) response element binding protein associated factor (pCAF) as shown in *in vitro* and *in vivo* mice studies to study ultraviolet radiation (UV)-induced skin damage [41,42]. In the current study, we used AA to induce histone de-acetylation *in vitro* and *ex vivo,* presumably via a similar mechanism. We show that AA can block Foxp3 binding to the IL-2 promoter and result in a concurrent increase in IL-2 mRNA levels *in vitro*. We also demonstrated that treatment of virus-specific CD8^+^ T cells of FIV-infected cats with AA for 24 h followed by co-culture with autologous Treg cells resulted in a reduction in Foxp3 binding at the IL-2 promoter. Taken together, these data suggest that histone acetylation contributes to Treg-induced Foxp3-mediated suppression of CD8^+^ T cell function during lentiviral infection. These results further imply that epigenetic modulations of dysfunctional CD8^+^ T cells could be employed to boost CD8^+^ T cell function and enhance the clearance of virally-infected cells in HIV cure strategies. 

## 2. Materials and Methods

### 2.1. Cats and FIV Infection

Specific pathogen-free (SPF) cats were obtained from Liberty Labs (Liberty Corners, NJ, USA) or Cedar River Laboratory (Charles City, IA, USA) and housed at the Laboratory Animal Resource Facility at the College of Veterinary Medicine, North Carolina State University. Cats (*n* = 5) were inoculated with 10^5^ TCID_50_ of FIV-NCSU_1_ intravenously. Feline immunodeficiency virus infection was confirmed by ELISA (*S*NAP FIV/FeLV; Idexx Laboratories, Greensboro, NC, USA). Samples were collected from cats at 6–12 months (chronic phase) post infection. All protocols were approved by the North Carolina State University Institutional Animal Care and Use Committee (protocol #12-176-B, 2013–2016). Single-cell suspensions were prepared from popliteal or submandibular peripheral lymph nodes (PLNs) obtained through surgical biopsies by gently and repeatedly injecting sterile CTL media (1640-RPMI with 2 mM l-glutamine (Thermo Fisher, Waltham, MA, USA), 10% fetal bovine serum (FBS) (HyClone, South Logan, UT, USA), 1% penicillin-streptomycin (Thermo Fisher), 1.5 g/L sodium bicarbonate (Sigma Aldrich, St. Louis, MO, USA), 10 mM (4-(2-hydroxyethyl)-1-piperazineethanesulfonic acid (HEPES) (Thermo Fisher) and 1.0 mM sodium pyruvate (Thermo Fisher) into the tissue using an 18 G needle until the cells were released from the tissue. Cell counts and viability were determined by Trypan Blue dye exclusion using the Luna II cell counter (Logos Biosystems, Anyang-si, South Korea), and viability of *ex vivo* cells was always found to be >90%.

### 2.2. CD8^+^T Cell Co-Culture and CFSE Cell Proliferation Assays

Both anti-feline CD4 and anti-feline CD8 monoclonal antibodies were developed by our feline lentivirus research group as described previously [43]. The feline anti-CD25 monoclonal antibody was developed by K. Ohno from University of Tokyo, as described previously [20]. Single cells from LNs were suspended at 1 × 10^8^ cells/mL in Hank’s Balanced Salt Solution (HBSS) (Thermo Fisher) with 2% FBS and stained with anti-feline CD8 PE antibody (clone 3.357) at 4 °C for 30 min. EasySep^®^ PE Selection Cocktail was added at 100 μL/mL of cell suspension at RT for 15 min, then EasySep^®^ Magnetic Nanoparticles were added at 50 μL/mL at RT for 10 min. CD8^+^PE^+^ cells were separated by using the magnet provided in the kit (Stem Cell, Vancouver, BC, Canada). The rest of the cell suspension was stained with mouse anti-feline CD4 APC antibody to isolate CD4^+^ cells by using EasySep^®^ APC Selection kit (Stem Cell). Isolated CD4^+^ cells were then stained with mouse anti-feline CD25 FITC antibody to sort CD4^+^ CD25^+^ double positive Treg cells using the MoFlo XDP high-speed cell sorter (Beckman Coulter, Brea, CA, USA). DAPI (BioLegend, San Diego, CA, USA) was used as the cell viability dye to ensure we obtained live cells at the end of each of the sorts. CD8^+^ T cells were resuspended in pre-warmed phosphate buffered saline (PBS) (Thermo Fisher) /0.1% bovine serum albumin (BSA) (Sigma Aldrich) and stained with 10 μM carboxyfluorescein succinimidyl ester (CFSE) dye from the Cell Trace^TM^ CFSE Cell Proliferation Kit (Life Technologies, Carlsbad, CA, USA). CD8^+^CFSE^+^ T cells were returned to lymph node (LN) culture without CD4^+^CD25^+^ Treg cells and stimulated *in vitro* with ultraviolet (UV)-inactivated FIV-NCSU1 for 72 h. Following stimulation, the virus specific proliferating CFSE^int/lo^ cells and non-specific CD8^+^ T cells CFSE^high^ were isolated by re-sorting using a high-speed cell sorter. For all the co-culture studies presented here, CD8^+^ lymphocytes were co-cultured at a 1:1 (Treg: CD8^+^) ratio with autologous CD4^+^CD25^+^ Treg cells for 24 h. After co-culture, the cells were washed and then resorted into CD8^+^ populations for analysis by qPCR or Chromatin immunoprecipitation (ChIP). The purity of magnetic bead sorted cells was >95% and Moflo XDP sorted cell populations was >99%.

### 2.3. Mya-1 Cell Culture and Cell Viability

Mya-1 feline CD4^+^ T cells were cultured in RPMI 1640 medium with 2 mM l-glutamine adjusted to contain 1.5 g/L sodium bicarbonate, 4.5 g/L glucose, 10 mM HEPES, 1.0 mM sodium pyruvate, 10% FBS, and 1% penicillin and streptomycin and supplemented with 0.05 mM 2-mercaptoethanol and 100 units/mL recombinant human IL-2 (R&D Systems, Minneapolis, MN, USA). Cultures were maintained by the addition of fresh medium to cells every 2–3 days, maintained at 37 °C in a humidified atmosphere containing 7% CO_2_. Cells were stained with Trypan Blue and counted with Luna II cell counter (Logos Biosystems) for cell viability assessment. The viability was always found to be ~80%. 

### 2.4. Chromatin Immunoprecipitation (ChIP) and Acetyl-Histone 3 (AcH3) ChIP Assay

The ChIP was performed using Chroma Flash High-Sensitivity ChIP Kit (Epigentek, Farmingdale, NY, USA) according to manufacturer’s specifications. In brief, 2 μg of anti-Foxp3 (Abcam, Cambridge, UK), anti-acetyl-histone 3 (Millipore, Burlington, MA, USA), anti-RNA polymerase II (positive control) and non-immune IgG (negative control) antibodies were first bound to assay strip wells. The sorted cells were cross-linked by adding CTL media containing formaldehyde to a final concentration of 1% with incubation at room temperature (20–25 °C) for 10 min on a rocking platform (50–100 rpm). To each tube, pre-warmed 1.25 M glycine (1:10) was added to a final concentration of 125 mM and incubated at room temperature for 5 min. After washing with ice-cold PBS, working lysis buffer was added to re-suspend the cell pellet and incubated on ice for 10 min. After carefully removing the supernatant, working ChIP buffer was added to re-suspend the chromatin pellet. Sonic Dismembrator 50 (Fisher Scientific, Hampton, NH, USA) was used to shear chromatin. The program was set to 25% power output and sonication was done with 4 pulses of 15 s each, with 30 s rest on ice between each pulse. Chromatin immunoprecipitation samples were centrifuged at 12,000 rpm at 4 °C for 10 min after shearing and the supernatant was transferred to a new vial. The ChIP samples were added to the wells coated with antibodies, positive control, or negative control. The reaction wells were incubated at 4 °C overnight. The samples were then washed according to the protocol and subjected to reverse cross-linking at 42 °C for 30 min, 60 °C for 45 min. DNA release was at 95 °C for 15 min in a thermocycler. Finally, the DNA samples were purified by spin column for quantitative PCR (qPCR) using custom designed ChIP primers for the IL-2 promoter: 

Forward primer: 5’-TGCTCCACATGTTCAACACA-3’, reverse primer: 5’-CCCACACTTAGGTGGCAGTT-3’. The feline IL-2 promoter sequence (3000 bp upstream of the transcription start site) was obtained from UCSC Genome Browser, primers were designed using NCBI Primer Blast. The primer sequences were validated by Sanger sequencing (Genewiz, Morrisville, NC, USA). Three experimental replicates per animal were performed during the PCR step. The relative enrichment of the target gene was calculated. Fold enrichment (FE) was calculated by using a ratio of amplification efficiency of the ChIP sample over that of non-immune IgG, FE % = 2^(NIgG Ct − Sample Ct)^ × 100% [22]. GAPDH amplification in RNA polymerase II-bound DNA was used as a control for PCR conditions. Ct values of non-immune IgG were used for input cell normalization.

### 2.5. RNA Extraction, RT and Real-Time PCR Quantification

Total RNA was extracted from cells using PureLink ^TM^ RNA Micro Kit (Life Technologies). The concentration was quantified using a Nano Drop Spectrophotometer (Thermo Fisher). RT PCR was performed for mRNA using qScript cDNA Synthesis Kit (Quanta Biosciences, Beverly, MA, USA). Reactions for cDNA synthesis (total volume—15 µL) were incubated for 5 min at 22 °C, 40 min at 42 °C, and 5 min at 85 °C to inactivate the reverse transcriptase. Feline specific primers as shown in Table 1 were used to detect the Foxp3 and IL-2 mRNA levels using LightCycler^®^ 480 System (Roche, Basel, Switzerland) qPCR. GAPDH mRNA expression was used as a normalizing control. Primers in Table 1 were designed by NCBI Primer Blast and validated by Sanger sequencing (Genewiz). For qPCR experiments, a ΔΔCt ratio was used to quantify relative mRNA expression. Each 20 µL reaction comprised of 8 µL of diluted cDNA, 10 µL PerfeCTa SYBR Green Super Mix Reaction Mix (Quanta Biosciences), 1 µL forward primer and 1 µL of reverse primer were run in triplicates under the following cycling conditions: hot start enzyme activation at 95 °C for 5 min, denatured at 94 °C for 45 s, annealed at 60 °C for 45 s, and elongated at 72 °C for 1 min with 35 cycles, and final extension at 72 °C for 10 min. We performed mRNA analysis only on *Mya-1* cells. We were unable to perform these analyses on *ex vivo* feline virus-specific CD8^+^ T cells due to limited tissue availability.

### 2.6. Anacardic Acid (AA) Treatment

**Figure 1:**
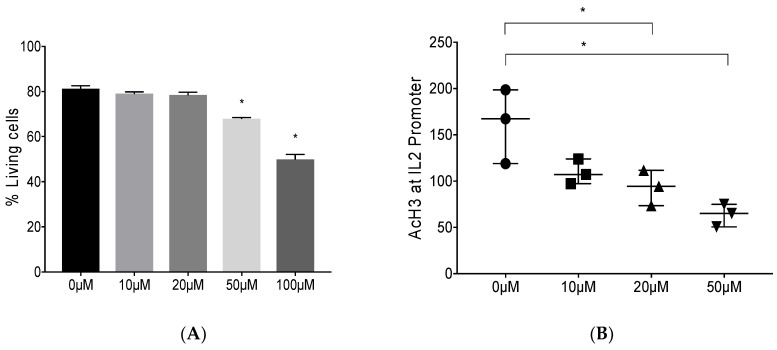
Epigenetic modulation by anacardic acid (AA) promotes histone de-acetylation. Feline Mya-1 cells, a CD4^+^ T cell line, were treated with anacardic acid (AA) at 0, 10, 20, 50, and 100 µM for 24 h. (**A**) Cell viability was measured by flow cytometry as described in the methods using DAPI as the live-dead stain. Data are represented as the mean+ SD, *n* = 3 (**B**). Acetylated Histone 3 (AcH3) chromatin immunoprecipitation (ChIP), followed by qPCR demonstrated a reduction in histone acetylation at the interleukin-2 (IL-2) promoter. Data are represented as the median with range, *n* = 3. Statistical differences were determined by paired *t*-test in (**A**) and Wilcoxon matched-pairs signed rank test in (**B**), with * representing *p* < 0.05.

Mya-1 cells (1 × 10^6^) cultured in 12-well plates were treated with 0 µM, 10 µM, 20 µM, and 50 µM of anacardic acid (Sigma Aldrich) for 24 h. Viability was measured by flow cytometry. DAPI (BioLegend) was used to stain the cells. The data was acquired on BD LSRII (Franklin Lakes, NJ, USA) and analyzed using FCS Expression version 6 software (https://www.denovosoftware.com/) when determining the appropriate concentration of AA (Figure 1A). For the rest of the experiments with AA treatment, Trypan Blue was used to determine viability and cells were counted with Luna II cell counter (Logos Biosystems). The viability was found to be ~80% for each experiment. 

### 2.7. Data Analysis

Statistical analysis was performed using GraphPad Prism software (San Diego, CA, USA). Samples of two different groups were compared by the Mann-Whitney test, and paired samples were analyzed by the Wilcoxon matched-pairs signed rank test. Viability data was analyzed using a paired t-test. *p* values of <0.05 were considered statistically significant. 

## 3. Results

### 3.1. Epigenetic Modulation by Anacardic Acid (AA) Promotes Histone De-Acetylation and Blocks Foxp3 Binding to the IL-2 Promoter Leading to Higher IL-2 mRNA Levels In Vitro

Based upon our previous studies modulating DNA methylation, we asked if reducing histone acetylation also blocks Foxp3 binding to the IL-2 promoter. Anacardic acid is reported to promote histone de-acetylation by inhibiting the activity of p300 histone acetyltransferase [44]. To determine the potential toxicity of AA, we first treated Mya-1 cells, a feline CD4^+^ T cell line, with increasing concentrations of AA (Figure 1A). At the same time, we measured histone 3 acetylation at the IL-2 promoter (Figure 1B). The results demonstrated that a 20 µM concentration of AA had limited toxicity to feline lymphocytes while promoting histone de-acetylation at the IL-2 promoter, and this concentration was selected to perform the subsequent experiments. 

**Figure 2:**
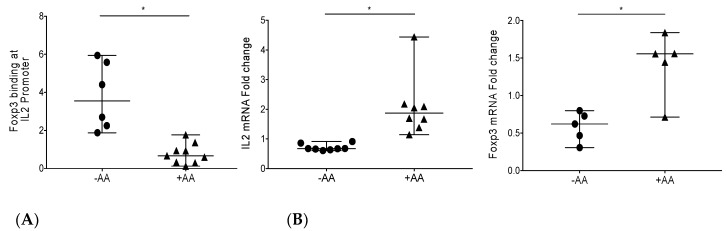
Anacardic acid blocks endogenous Foxp3 binding to the IL-2 promoter and increases IL-2 mRNA levels *in vitro*. (**A**) Mya-1 cells were either untreated (circles, *n* = 6) or treated with 20 μM AA (triangles, *n* = 9); AA treatment inhibited Foxp3 binding at the IL-2 promoter. (**B**) Mya-1 cells were either untreated (circles) or treated with 20 μM AA (triangles) and assessed by RT-qPCR. Both IL-2 mRNA (*n* = 8) and forkhead box P3 (Foxp3) mRNA (*n* = 5) were increased following AA treatment. All data are presented as the median with range, where each point represents an individual experiment. Statistical differences were determined by Mann-Whitney test, with * representing *p* < 0.05.

To determine if AA blocks the binding of the repressive transcription factor Foxp3 to the IL-2 promoter, feline Mya-1 cells were either untreated or treated with 20 µM AA for 24 h. Chromatin immunoprecipitation followed by qPCR demonstrated that 20 μM AA treatment significantly inhibited endogenous Foxp3 binding to the IL-2 promoter (Figure 2A). To validate the biological significance of this finding, we investigated the effect of AA on the transcription of IL-2. qPCR data confirmed that IL-2 mRNA levels increased with 20 μM AA treatment (Figure 2B). Foxp3 mRNA levels were also enhanced by AA treatment and, therefore, reduced Foxp3 binding to the IL-2 promoter was not due to reduced Foxp3 transcription in AA-treated Mya-1 cells (Figure 2B). Collectively these results demonstrated that AA blocks Foxp3 binding to the IL-2 promoter concomitant with a reciprocal increase in IL-2 transcription *in vitro*. 

### 3.2. Anacardic Acid Blocks Foxp3 Binding to the IL-2 Promoter In Virus-Specific CD8^+^ T Cells Co-Cultured with Autologous Treg Cells in FIV+ Cats

**Figure 3:**
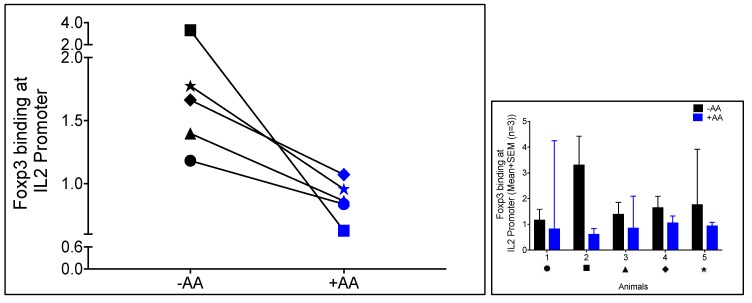
Anacardic acid blocks Foxp3 binding to the IL-2 promoter in virus-specific CD8^+^ T cells. Virus-specific CD8^+^ lymphocytes from FIV+ cats (>6 mo infection) were either untreated (black) or treated (blue) with AA at 20 µM for 24 h followed by co-culture with autologous Treg cells. Foxp3 ChIP followed by qPCR demonstrated a reduction in Foxp3 binding to the IL-2 promoter in each paired set (*n* = 5, each symbol represents an individual animal, values for each animal in the left panel represents the average of three technical replicates). The panel on the right demonstrates Foxp3 binding at the IL-2 promoter in virus-specific CD8^+^ T cells for each animal (symbol under X-axis) in the untreated (black) and AA treated (blue) groups. Data is presented as the mean + SEM of three technical replicates.

The potential importance of this epigenetic modification *in vivo* during lentiviral infection was assessed by treating virus-specific CD8^+^ T cells from FIV-infected cats with or without 20 µM AA for 24 h, followed by co-culture with autologous Treg cells. Subsequently, the CD8^+^ T cells were re-isolated for Foxp3 ChIP followed by qPCR for the IL-2 promoter. Consistent with our *in vitro* findings using Mya-1 cells, Foxp3 binding to the IL-2 promoter was decreased when virus-specific CD8^+^ T cells were pre-treated with AA (Figure 3). Although the difference in Foxp3 binding to the IL-2 promoter between treated and untreated CD8^+^ T cells did not reach statistical significance (*p* = 0.08), likely due to the low sample number, reduced Foxp3 binding was observed in each paired sample set, indicative of biological significance. 

## 4. Discussion

Our research group and others have reported that feline immunodeficiency virus (FIV)-infected T regulatory (Treg) cells induce forkhead box P3 (Foxp3) expression in activated CD8^+^ T cells via a tumor growth factor-β (TGF-β)-dependent mechanism. We also demonstrated that Foxp3 binds the interleukin-2 (IL-2), interferon-γ (IFN-γ), and tumor necrosis factor-α (TNF-α) promoters in virus-specific CD8^+^ T cells with a concomitant decrease in the mRNA levels of the cytokines alluding to the suppressor function of Foxp3, and that blocking DNA de-methylation blocks Foxp3 binding to the IL-2 promoter [20,21,22,25]. These results suggest that changes in DNA methylation, in part control Foxp3 binding to the IL-2 promoter during FIV infection. In concert with our previous findings, a recent report demonstrates Foxp3 binding to gene enhancer regions [45]. Therefore; we have continued to investigate the effects of altered chromatin availability via epigenetic modifications, such as histone acetylation, on Foxp3 binding to gene promoters. In the current study, we investigated whether modulation of histone acetylation altered Foxp3 binding to the IL-2 promoter. The histone H3 tail is susceptible to different covalent modifications such as acetylation, phosphorylation, and methylation of lysine and serine residues, thereby regulating gene activation or suppression. Histone acetylation has been related to gene activation and has been shown to induce a transcriptionally active chromatin [46]. Therefore, we hypothesized that reducing histone acetylation might also prevent Foxp3 binding. To address our hypothesis, we tested whether anacardic acid (AA), a known histone acetyltransferase (HAT) inhibitor inhibits Foxp3 binding to the IL-2 promoter [44]. Consistent with the function of AA as a HAT inhibitor, we demonstrate that AA treatment reduced histone 3 acetylation in the feline cell line Mya-1 (Figure 1B). Further, AA treatment of Mya-1 cells reduced Foxp3 binding to the IL-2 promoter (Figure 2A). Foxp3 transcription itself is epigenetically regulated (reviewed in Li et al.) [19,47]. *In vitro* and *in vivo* studies in mice have shown that HAT p300 drives Foxp3 acetylation and directly affects Treg suppressive activity [48,49]. Therefore, there might be direct impact of AA (p300 inhibitor) on Foxp3 acetylation levels, as well, which were not considered in the study. IL-2 mRNA levels were higher in AA-treated versus untreated Mya-1 cells further supporting our model of Foxp3-mediated suppression (Figure 2). Foxp3 can either act as an active or a passive repressor. While acting as a passive repressor, it turns down the enhancer activity to reduce the positive effect of the enhancer whereas when acting as an active repressor, it binds with repressor complexes, such as histone methyltransferase enhancer for zeste homolog 2 (EZH2), and transcription factors Yin Yang 1 (YY1) and IKAROS family zinc finger 3 (IKZF3) [29,30,50]. While we focus on the suppressor function of Foxp3 during FIV infection in this article, it is important to note that Foxp3 can have dual repressor and activator functions depending on the genes and transcription factors it interacts with. Foxp3 cooperates with transcription factor P65 (RelA), IKAROS family zinc finger 2 (IKZF2), and histone acetyltransferase KAT5 to function as a transcriptional activator [30,47,51].

Previous studies have shown that the distal enhancer region of the IL-2 gene undergoes increased H3 acetylation upon activation, and that the IL-2 promoter at the proximal region exhibits decreased histone acetylation due to the loss of H3 and H4 proteins in response to T cell activation [37,38]. We and others have demonstrated that CD8^+^ T cells exhibit an activated phenotype during FIV infection [20,52]. Here, we describe a potential role for histone acetylation in lentiviral infection by demonstrating the reduction in Foxp3 binding to the IL-2 promoter upon AA treatment of FIV-specific CD8^+^ T cells isolated from each of the five animals (Figure 3). Even though this dataset was not significant (*p* = 0.08), a consistent reduction in Foxp3 binding to the IL-2 promoter after AA treatment was observed for every animal suggesting the biological relevance. Therefore, the lack of significance was most likely due to the small sample size (*n* = 5). There was high variability within the data for AA treated group for animal 1 and untreated group for animal 5 which also could have impacted the overall significance in difference between the treated and untreated groups. Overall, this data suggests that epigenetic modulation of dysfunctional CD8^+^ T cells during lentivirus infection can reduce Foxp3 binding. Future studies are needed to evaluate whether AA could be applied *in vivo* to restore, or at a minimum, enhance the function of antiviral CD8^+^ T cells at different stages of FIV infection. 

Here our primary focus was to investigate the effect of inhibiting histone acetylation via AA. A recent study demonstrated the p300 HAT suppressive function of AA to reduce ultraviolet (UV)-induced histone modifications in hairless mice *in vivo* [42]. However, AA has multiple mechanisms of action that might have affected our results. Anacardic acid activates aurora kinase A (ARK) and mediates phosphorylation of histone H3 *in vitro*, it also inhibits post-transcriptional modifications, such as sumoylation [53]. A recent study reported a reduction in the DNA methylation levels at the collagen type 1 alpha 2 chain (COL1A2) promoter region upon treatment with AA suggesting its role as a DNA methyltransferase inhibitor [54]. The anti-proliferative functions of AA have been demonstrated in breast, prostate, and lung cancer cells, except for in ovarian cells where it promotes proliferation and metastasis [44,55,56,57]. Treatment with AA also suppresses the NF-KB pathway and reduces TNF-α and IL-6 levels, supporting an anti-inflammatory milieu [40,42]. Therefore, “off-target” effects of AA cannot be ruled out. Despite these limitations, to our knowledge, this is the first account indicating the involvement of histone acetylation, in the suppression of IL-2 in virus-specific CD8+ T cells using the FIV model. This study prompts a new area of investigation to advance our understanding of virus-specific CD8^+^ T cell dysfunction and avenues to boost CD8^+^ T cell function for potential cure strategies. 

Based upon our findings presented here, we propose that the following occurs: lentivirus-induced activation of virus-specific CD8^+^ T cells results in chromatin remodeling as these T cells upregulate the expression of genes essential to antiviral function. During this process, histone acetyltransferases add acetyl groups to histone tails, relaxing the chromatin to its euchromatin form, and DNA becomes accessible for transcription factors to bind. Collectively the results presented here suggest that the potential exists to de-acetylate histones and prevent Foxp3 binding to the IL-2 promoter *in vivo* during the course of lentiviral infection. The reduction in Foxp3 binding at the IL-2 promoter may translate into restoring or enhancing IL-2 function in the dysfunctional CD8^+^ T cells during lentivirus infections. 

## Figures and Tables

**Table 1:** List of primers used for qPCR.

Primer Target	Forward	Reverse
Foxp3	5’-GCCTGCCACCTGGAATCAAC-3’	5’-GTGTGCTGGGGCTTGGGA-3’
IL-2	5’-ACAGTGCACCTGCTTCAAGCTCT-3’	5’-CCTGGAGAGTTTGGGGTTCTCAGG-3’
GAPDH	5’-GGAGAAGGCTGGGGCTCAC-3’	5’GGTGCAGGAGGCATTGCTGA-3’
